# Optimal timing for lung metastasectomy in patients with colorectal cancer

**DOI:** 10.1093/icvts/ivac224

**Published:** 2022-08-22

**Authors:** Junji Ichinose, Kohei Hashimoto, Yosuke Matsuura, Masayuki Nakao, Takashi Akiyoshi, Yosuke Fukunaga, Sakae Okumura, Mingyon Mun

**Affiliations:** Department of Thoracic Surgical Oncology, Cancer Institute Hospital of Japanese Foundation for Cancer Research, Tokyo, Japan; Department of Thoracic Surgical Oncology, Cancer Institute Hospital of Japanese Foundation for Cancer Research, Tokyo, Japan; Department of Thoracic Surgical Oncology, Cancer Institute Hospital of Japanese Foundation for Cancer Research, Tokyo, Japan; Department of Thoracic Surgical Oncology, Cancer Institute Hospital of Japanese Foundation for Cancer Research, Tokyo, Japan; Department of Colorectal Surgery, Cancer Institute Hospital of Japanese Foundation for Cancer Research, Tokyo, Japan; Department of Colorectal Surgery, Cancer Institute Hospital of Japanese Foundation for Cancer Research, Tokyo, Japan; Department of Thoracic Surgical Oncology, Cancer Institute Hospital of Japanese Foundation for Cancer Research, Tokyo, Japan; Department of Thoracic Surgical Oncology, Cancer Institute Hospital of Japanese Foundation for Cancer Research, Tokyo, Japan

**Keywords:** Pulmonary metastasis, Surgery, Optimal timing, Colorectal cancer

## Abstract

**OBJECTIVES:**

The possibility of occult metastasis remains a concern when deciding on lung metastasectomy. This study aimed to evaluate the utility of our two-step determination, which required confirmation that no new metastases had occurred over 3 months before surgery.

**METHODS:**

Patients who were referred for colorectal lung metastases between 2007 and 2015 were reviewed. Immediate wedge resection was performed for cases with a single peripheral metastasis, whereas surgical indications for others were determined by the two-step determination. Early increase was defined as the emergence of new metastases within 4 months after the diagnosis of lung metastases.

**RESULTS:**

Among 369 patients included, 92 were unresectable upon initial diagnosis, and 74 with single peripheral metastasis underwent immediate wedge resection. Surgical indications for the remaining 203 patients were ascertained based on the two-step determination. Surgery was not indicated in 48 patients (24%) due to new metastases or a favourable response to chemotherapy, with a median waiting duration of 4.8 months. Those who did not receive surgery had a worse prognosis than those who did (5-year overall survival: 21% vs 69%, *P < *0.001) and were comparable to the initially unresectable group (5-year overall survival: 23%). Thirty-eight patients with early increase had lower surgical resection rates and worse prognoses than those without. Multivariable analysis identified early increase as an independent prognostic factor (hazard ratio: 4.49, *P < *0.001).

**CONCLUSIONS:**

Patients with colorectal lung metastasis who developed new metastasis during the waiting period exhibited poor prognosis, suggesting the utility of the two-step determination of surgical indications.

**Figure:**
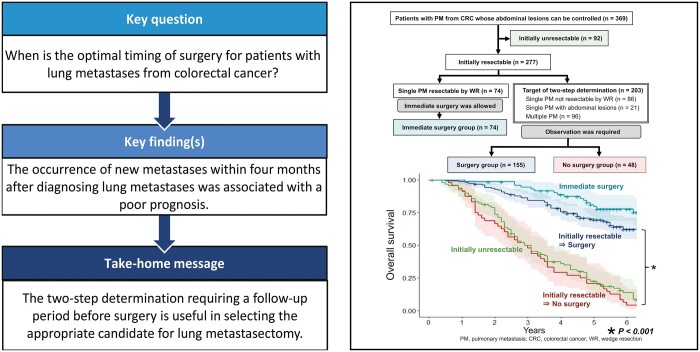

## INTRODUCTION

The lung has been reported to be the second most common organ after the liver to which colorectal cancer (CRC) metastasizes, with 10–15% of CRC cases having pulmonary metastases (PM) [1]. Numerous case series and systematic reviews have shown that pulmonary metastasectomy for CRC was associated with improved long-term survival in selected patients, with a 5-year overall survival (OS) of 40–60% [2–4], and it has been considered a practical standard treatment option [5].

However, advancements in chemotherapy and targeted therapy have recently improved the survival rate of patients with metastatic CRC. Current pulmonary metastasectomy is part of a multidisciplinary treatment process; thus, it requires careful determination of whether surgery or chemotherapy should be performed using advanced diagnostic imaging techniques. The only randomized controlled trial that compared lung metastasectomy and continued active monitoring (PulMiCC trial) [6] revealed that the estimated OS in the metastasectomy and active monitoring groups was 38% and 29%, respectively, with no significant difference between both groups. A multicentre trial is ongoing in which patients with PM from CRC are divided into low- and high-risk groups. In this trial, low-risk patients were treated by surgical resection, and recurrence-free survival was compared with and without perioperative chemotherapy, whereas high-risk patients were treated by systemic chemotherapy, and OS was compared with and without surgical resection [7]. The most crucial point in the local treatment of PM is assessing the disease biology and selecting appropriate candidates; futile surgery for patients who are unexpected to be cured should be avoided.

The possibility of clinically occult metastases remains a substantial concern when deciding on surgical intervention for resectable PM of CRC. By the time PM is diagnosed, cancer would have progressed into a systemic disease, leading to small occult metastases in the lungs and other organs that are undetectable via diagnostic imaging. Unfortunately, following current diagnostic imaging techniques, the only method to detect these small lesions is to wait for them to grow to a detectable size. Therefore, our group has established a method to confirm no increase in lesions over 3 months before surgery except for cases with a single peripheral metastasis that can be removed by wedge resection. This study evaluated the utility of our two-step determination of surgical indication. Furthermore, this study investigated the relationship between the time since PM diagnosis and the cumulative incidence of new metastases, as well as the prognostic impact of the early development of new metastases.

## PATIENTS AND METHODS

### Ethics statement

This study was approved by the Institutional Review Board for Clinical Research of the Cancer Institute Hospital on 21 May 2021 (approval no. 2021-1020) and was conducted following the principles of the Declaration of Helsinki and its later amendments. The institutional review board waived the requirement for informed consent due to the retrospective nature of the study.

### Study design and patient cohort

This is a retrospective, observational study. This study reviewed patients referred to our department for PM from CRC between 2007 and 2015. Chest computed tomography (CT) was obtained at our hospital during regular check-ups before and after treatment for CRC to determine PM presence. If a suspected PM lesion was discovered, the patient was referred to our department for possible resection. Experienced thoracic surgeons and radiologists clinically diagnosed PM from CRC based on chest CT images. Patients who were pathologically diagnosed with tumours other than PM from CRC were excluded. Pulmonary resection was indicated when complete resection was possible, no thoracic lymph node involvement existed and all extrathoracic metastases were resected. The presence of thoracic lymph node and extrathoracic metastases was mainly determined using CT and positron emission tomography; biopsy, such as endobronchial ultrasound-guided transbronchial needle aspiration, was performed when necessary. Although no limit was imposed on the number of PM to be resected, those that required pneumonectomy or bilateral lobectomy were regarded as contraindications.

### Two-step determination of surgical indication

Patients considered eligible for resection at initial diagnosis were divided into 2 groups: those with a single peripheral metastasis and others. Immediate surgery was recommended for cases with a single peripheral metastasis that can be removed by wedge resection. Surgical indications for the others were determined by two-step determination: surgery was performed after confirming that the number of lesions did not increase during at least 3 months of follow-up. If the number of lesions increased during that period, the patient was followed up for another 3 months. If there was no further increase in the number of lesions, surgery was recommended. Chemotherapy was provided on a case-by-case basis during the follow-up period, following a multidisciplinary conference attended by surgeons and medical oncologists. When chemotherapy was highly effective such that lung metastatic lesions shrunk to an extent where they were difficult to identify during surgery, the patient was deemed unfit for surgery. To verify the effectiveness of this two-step determination of surgical indication, patients who underwent surgery and those deemed ineligible for surgery after follow-up were compared based on OS. The date of PM diagnosis was defined as the date at which a nodule suspected to be PM was first noted following CT or the date at which a nodule with a maximum diameter of ≥5 mm was identified in the same location following CT after reviewing previous CTs.

### Appropriate observation period

The relationship between time since diagnosis and the cumulative incidence of new metastasis was calculated to determine the appropriate follow-up period. After PM diagnosis, chest and abdomen CTs were performed every 3–6 months. Cases showing any signs or symptoms of recurrence were subjected to further evaluation, including abdominal magnetic resonance imaging and positron emission tomography. Early increase was defined as the emergence of new metastases within 4 months after PM diagnosis, and its impact on prognosis was examined. The early increase did not account for the growth in tumour size. The relationship between early increase and clinical characteristics, as well as factors predicting early increase, was also investigated.

### Statistical analysis

Statistical analyses were performed using IBM SPSS Statistics v.25 software (SPSS Inc., Chicago, IL, USA) or R v.4.0.3 (R Foundation for Statistical Computing, Vienna, Austria). Continuous variables were expressed as average ± standard deviation. Variables were compared and analysed using Student’s *t*-test, Welch’s method or χ^2^-test. OS was calculated starting from the date of PM diagnosis to the time of death from any cause using the Kaplan–Meier method and compared using the log-rank test. The Kaplan–Meier method was used to estimate the cumulative incidence rate of new metastasis. Competing risk analysis was considered unnecessary given the lack of patients who died from other causes within 2 years after PM diagnosis. Cox proportional hazard regression model was used for multivariable analyses to determine factors independently associated with OS. We used a stepwise backward elimination method to remove variables with a *P*-value of >0.1. Factors predicting early increase were examined using logistic regression analysis. *P*-values <0.05 indicated statistical significance.

## RESULTS

### Decision on treatment plan

**Figure 1:**
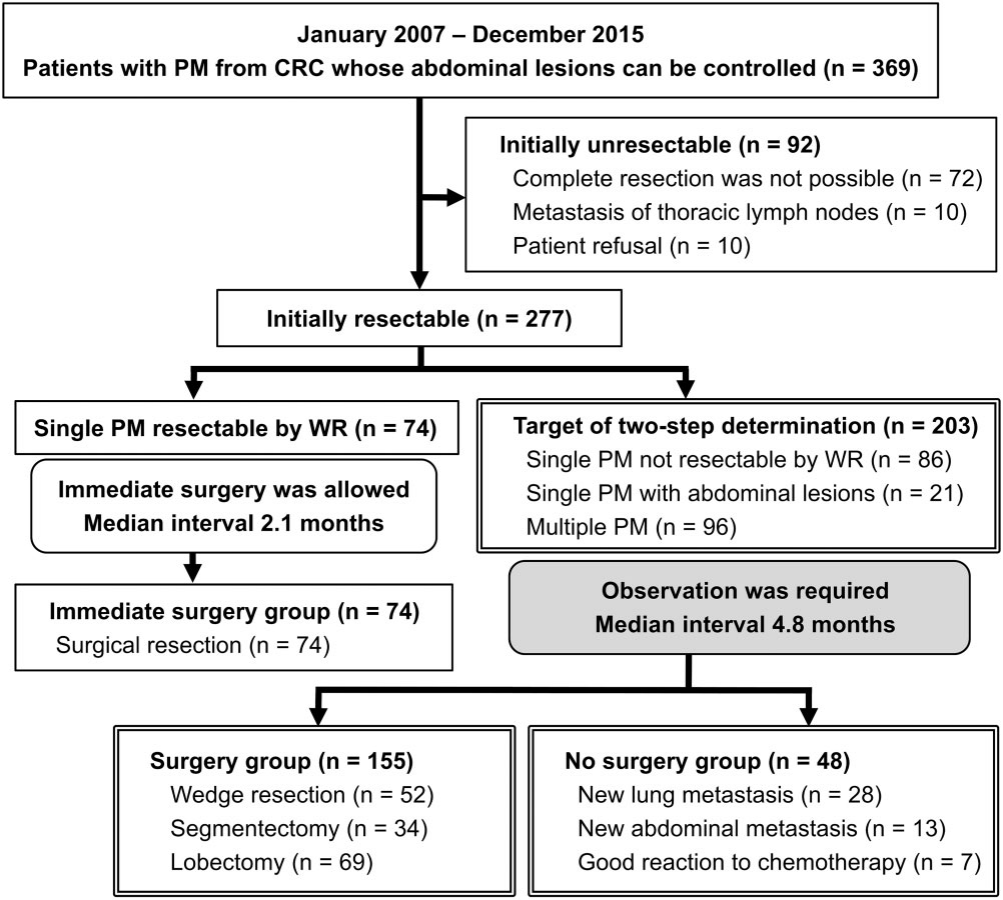
The patient flow diagram. CRC: colorectal cancer; PM: pulmonary metastasis; WR: wedge resection.

Figure 1 depicts the patient flow diagram. This study reviewed 369 patients referred to our department for PM from CRC between 2007 and 2015. There was no patient with incomplete data. Surviving patients were followed up for a median duration of 6.1 years. Among the 369 patients with PM from CRC referred to our department for controllable abdominal lesions, 92 (25%) were deemed ineligible for surgery upon initial diagnosis due to the impossibility of complete resection (72 cases), intrathoracic lymph node metastasis (10 cases) and patient refusal (10 cases). Two of the patients who refused surgery received radiation therapy. Immediate wedge resection was performed in 74 cases with a single PM (median interval from diagnosis of 2.1 months). All patients underwent surgery, and only 2 exhibited early relapse within 6 months of diagnosis. The immediate surgery group had a favourable prognosis (5-year OS, 79%).

Surgical indications for the remaining 203 patients were ascertained based on a two-step determination (median waiting period of 4.8 months). One hundred fifty-five patients underwent surgery (52 wedge resections, 34 segmentectomies and 69 lobectomies), whereas 48 cases did not undergo surgery due to new PM (28 patients), new abdominal lesions (13 patients) and favourable response to chemotherapy (7 patients). There were no cases in which surgery was not indicated due to an increase in tumour size or the development of intrathoracic lymph nodes, brain or bone metastases. In the 155 patients treated locally, a median waiting period of 4.5 months resulted in a slight increase in maximum tumour diameter (from 1.2 ± 0.8 to 1.6 ± 0.9 cm). Thoracoscopy was performed in 83% of the surgery group, and most patients with bilateral PM underwent one-stage bilateral thoracoscopy.

### Results of two-step determination

**Figure 2:**
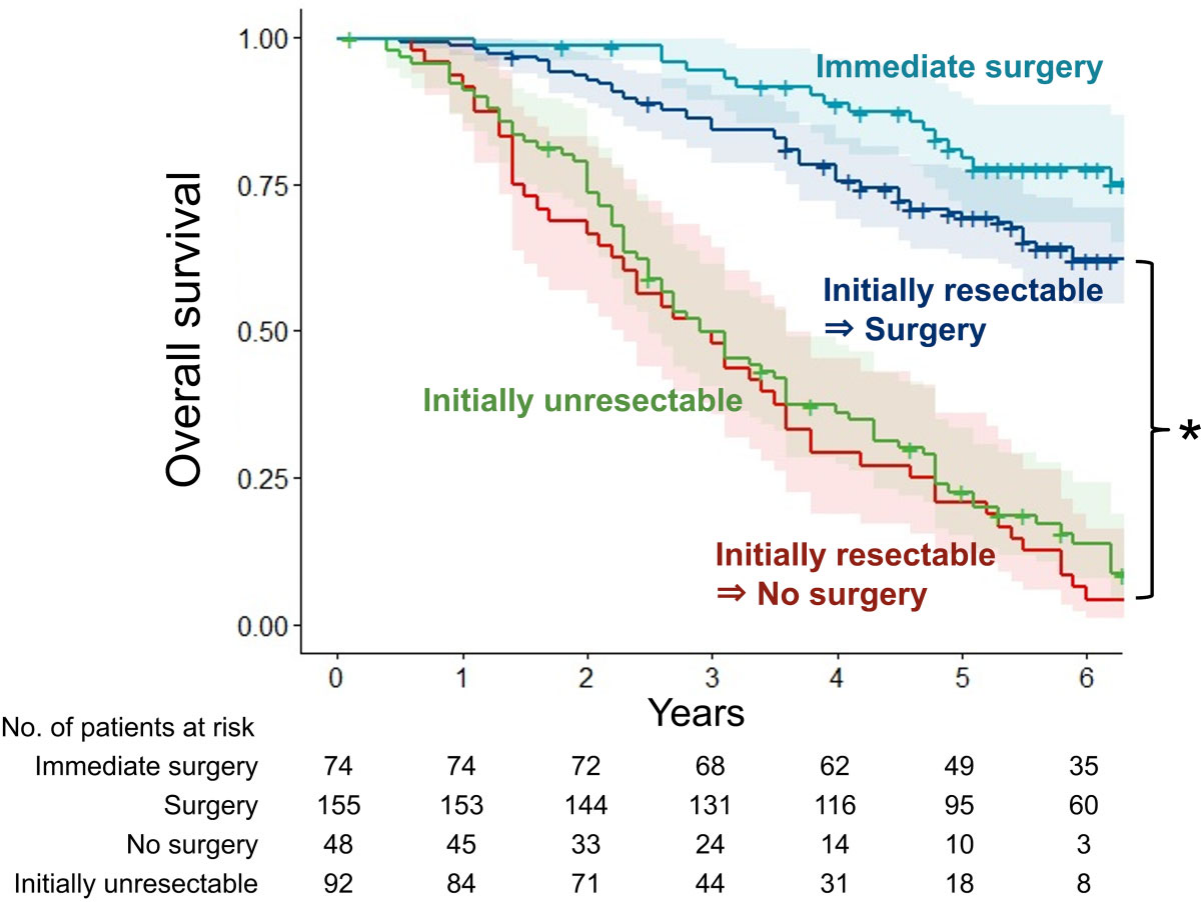
Overall survival curves after diagnosis in the surgery, no-surgery, immediate surgery and initially unresectable groups. Shaded areas represent the 95% confidence intervals. *P < 0.001.

Table 1 shows the characteristics of 203 patients who were targeted for a two-step determination. The number of PM at diagnosis was 1.7 ± 1.1 and 2.9 ± 1.8 in those who underwent surgery and those who did not, respectively, with a significant difference (*P < *0.001). Those who did not undergo surgery had smaller tumour sizes (1.0 ± 0.5 vs 1.2 ± 0.8 cm, *P = *0.042), more cases with extrathoracic metastasis (46% vs 26%, *P = *0.008), chemotherapy during the waiting period (75% vs 28%, *P < *0.001) and significantly shorter interval from the last local therapy (0.6 ± 0.6 vs 1.3 ± 1.8 months, *P < *0.001), compared with those who did. Moreover, patients who were unresectable after the observation period had a significantly poorer prognosis than those who underwent surgery (5-year OS, 21% vs 69%, *P < *0.001), which was similar to that of patients who were unresectable at initial diagnosis (5-year OS, 23%) (Fig. 2). The prognosis of those who did not undergo surgery was then examined according to contraindications. Accordingly, 7 patients who responded to chemotherapy had a 5-year OS of 71%, similar to that of those who underwent surgery, whereas 41 patients who developed new metastases during the waiting period had a 5-year OS of 12%, which was extremely poor.

**Table 1:** Characteristics of patients targeted for the two-step determination

	Surgery group	No-surgery group	*P*-Value
	(*n* = 155)	(*n* = 48)	
Age (years)	63 ± 10	62 ± 12	0.891
Sex: male, *n* (%)	89 (57)	22 (46)	0.159
Number of PM	1.7 ± 1.1	2.9 ± 1.8	<0.001
Tumour size (cm)	1.2 ± 0.8	1.0 ± 0.5	0.042
Simultaneous PM, *n* (%)	38 (25)	14 (29)	0.519
Past extrathoracic metastasectomy, *n* (%)	47 (30)	16 (33)	0.694
Presence of extrathoracic metastasis at diagnosis of PM, *n* (%)	40 (26)	22 (46)	0.008
Interval b/w colorectal surgery and diagnosis of PM (years)	1.7 ± 2.0	1.1 ± 1.5	0.077
Interval b/w the last local therapy and diagnosis of PM (years)	1.3 ± 1.8	0.6 ± 0.6	<0.001
Chemotherapy before diagnosis of PM, *n* (%)	84 (54)	26 (54)	0.997
Chemotherapy within 6 m after diagnosis of PM, *n* (%)	44 (28)	36 (75)	<0.001
Primary tumour location			0.595
Rectum, *n* (%)	89 (57)	27 (56)	
Left-side colon, *n* (%)	46 (30)	12 (25)	
Right-side colon, *n* (%)	20 (13)	9 (19)	
Nodal metastasis of primary lesion, *n* (%)	89 (57)	33 (69)	0.161

Continuous variables were expressed as average ± standard deviation.

b/w: between; PM: pulmonary metastasis.

### Appropriate follow-up period

**Figure 3:**
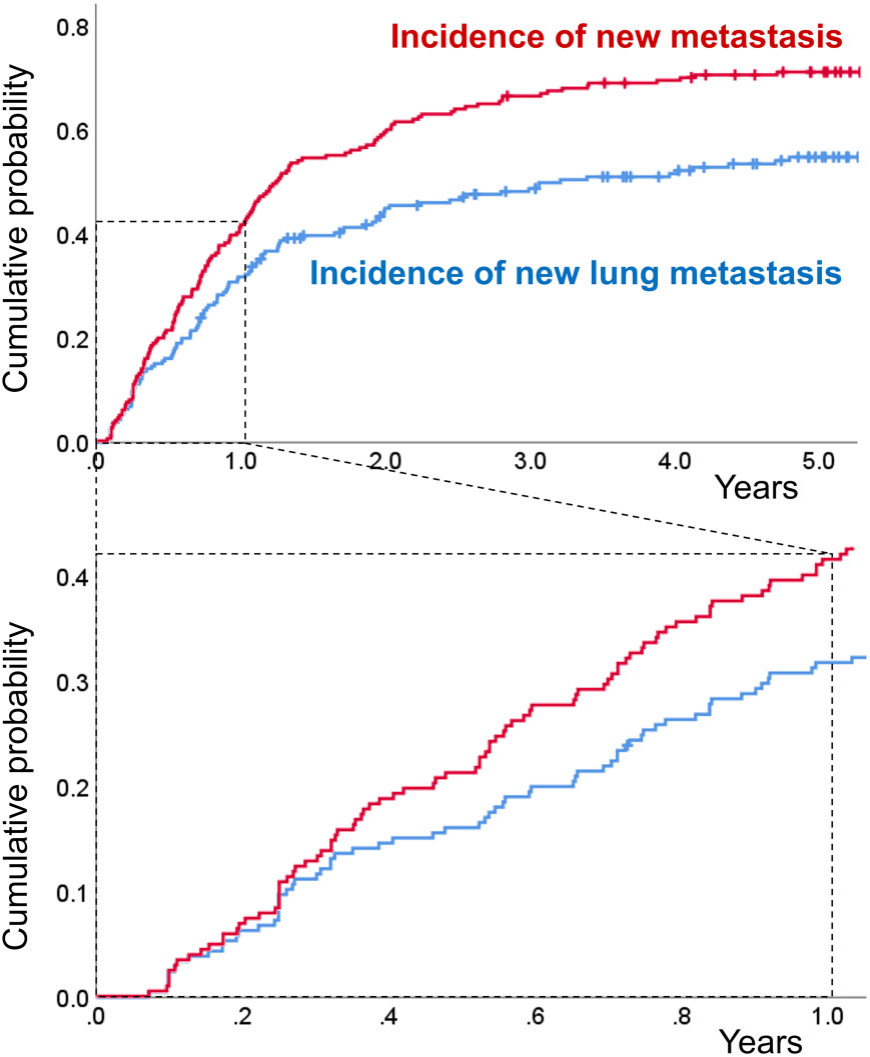
Cumulative probability of the incidence of new metastasis and new lung metastasis. CRC: colorectal cancer; PM: pulmonary metastasis; WR: wedge resection.

The cumulative probabilities for the incidence of new metastasis and new lung metastasis are summarized in Fig. 3. Accordingly, the incidence of new metastases increased linearly during the first year after diagnosis (i.e. 11%, 21% and 41% at 3 months, 6 months and 1 year, respectively). Thereafter, the rate of increase gradually slowed down, with 59% and 65% of cases being new metastases at 2 and 3 years, respectively. The incidence of new lesions limited to lung metastasis also increased in a similar manner.

Thirty-two patients who developed new metastasis within 4 months after PM diagnosis were defined as the early increase group. Differences in background factors between the early increase group and the rest of the patients are presented in Table 2. Accordingly, the early increase group was older (66 ± 10 vs 62 ± 10 years, *P = *0.036), had a higher rate of chemotherapy administration before PM diagnosis (78% vs 50%, *P = *0.003) and had fewer cases with simultaneous PM (9% vs 29%, *P = *0.022), compared with the rest of the patients. The presence or absence of chemotherapy during the flush-out period after PM diagnosis did not significantly correlate with the early increase. The early increase group had fewer patients who received surgery (47% vs 82%, *P < *0.001) and significantly poorer prognosis (5-year OS, 23% vs 65%, *P < *0.001) than the no early increase group. Among the patients in the early increase group, 15 who eventually underwent surgery had a 5-year OS of 37%, whereas 17 who did not undergo surgery had a considerably poor prognosis (5-year OS of 12%). Multivariable analysis identified early increase as an independent prognostic factor (hazard ratio: 4.49, *P < *0.001), along with the presence of extrathoracic metastases as revealed by PM diagnosis, chemotherapy during the observation period and metachronous PM (Table 3). Furthermore, multivariable logistic regression analysis identified multiple PM as a predictor of early increase (odds ratio: 2.86, *P = *0.019).

**Table 2:** Characteristics of patients who developed new metastasis within 4 months after diagnosis

	Early increase group	No early increase group	*P*-Value
	(*n* = 32)	(*n* = 171)	
Age (years)	66 ± 10	62 ± 10	0.036
Sex: male, *n* (%)	20 (63)	91 (53)	0.333
Number of PM	2.3 ± 1.5	1.9 ± 1.4	0.178
Tumour size (cm)	1.0 ± 0.7	1.2 ± 0.8	0.418
Simultaneous PM, *n* (%)	3 (9)	49 (29)	0.022
Past extrathoracic metastasectomy, *n* (%)	13 (41)	50 (29)	0.201
Presence of extrathoracic metastasis at diagnosis of PM, *n* (%)	7 (22)	55 (32)	0.246
Interval b/w colorectal surgery and diagnosis of PM (years)	1.5 ± 1.2	1.5 ± 2.0	0.846
Interval b/w the last local therapy and diagnosis of PM (years)	0.9 ± 0.7	1.2 ± 1.8	0.135
Chemotherapy before diagnosis of PM, *n* (%)	25 (78)	85 (50)	0.003
Chemotherapy within 6 m after diagnosis of PM, *n* (%)	9 (28)	71 (42)	0.155
Primary tumour location			0.391
Rectum, *n* (%)	16 (50)	100 (58)	
Left-side colon, *n* (%)	9 (28)	49 (29)	
Right-side colon, *n* (%)	7 (22)	22 (13)	
Nodal metastasis of primary lesion, *n* (%)	22 (69)	100 (59)	0.276
Local therapy for PM, *n* (%)	15 (47)	140 (82)	<0.001

b/w: between; PM: pulmonary metastasis.

Continuous variables were expressed as average ± standard deviation.

**Table 3:** Factors predicting overall survival after PM diagnosis from colorectal cancer analysed using Cox proportional hazard regression model

	Hazard ratio	95% CI	*P*-Value
Early increase, yes	4.49	2.73–7.38	<0.001
Simultaneous PM, yes	0.31	0.17–0.56	<0.001
Presence of extrathoracic metastasis at diagnosis of PM, yes	4.00	2.58–6.19	<0.001
Chemotherapy within 6 m after diagnosis of PM, yes	2.08	1.38–3.11	<0.001
Interval b/w the last local therapy and diagnosis of PM (years)	0.82	0.64–1.06	0.127

b/w: between; CI: confidence interval; PM: pulmonary metastasis.

## DISCUSSION

In this study, a two-step determination of surgical indications was made in 203 of the 277 patients who were considered resectable at the time of initial diagnosis, excluding 74 patients who underwent immediate wedge resection. Of those 203 patients, 48 (24%) were deemed ineligible for surgery after follow-up and had an extremely poor prognosis. Furthermore, the emergence of new metastases within 4 months of diagnosis was discovered to be an independent poor prognostic factor.

Although studies had identified the number of PM, intrathoracic lymph node metastasis, disease-free interval and presence of extrathoracic metastasis as prognostic factors [2–4, 8, 9], detailed criteria for surgical indications for PM from CRC vary by institution. Upon developing into PM, CRC can already be considered a systemic disease, with expectedly high recurrence rates even after complete resection. Among the patients included in this study, 65% developed new metastasis 3 years after PM diagnosis. As such, the efficacy of surgery should be carefully evaluated before deciding on surgical treatment. Moreover, considering that chemotherapy is expected to be effective in CRC, highly invasive surgery that does not allow for sufficient chemotherapy at the time of recurrence should be avoided. Based on the aforementioned concept, patients who had PM with intrathoracic lymph node metastasis and PM requiring pneumonectomy or bilateral lobectomy were considered ineligible for surgery.

Despite the clinical importance of the appropriate timing of lung metastasectomy, only a few studies have comprehensively examined this matter. Tanaka *et al.* [10] reported that patients who underwent lung metastasectomy within 3 months after diagnosis had a worse prognosis. Yamada *et al.* [11] reported that surgery within 9 months after diagnosis was a poor prognostic factor in patients with PM from CRC. However, these studies were retrospective in nature and included only surgical patients. Moreover, since the observation period had not been set based on established policies, potential bias cannot be ruled out (e.g. patients with smaller and slower-growing tumours tended to be observed for longer periods). This study examined 203 cases whose surgical indication was determined using a defined strategy, including cases in which surgery was not performed. After the observation period, 24% of patients who were thought to be resectable were deemed ineligible for surgery. These patients had a prognosis as poor as those who were initially unresectable. If the PM in this group had been resected based on the initial determination, their prognosis would have even been worse, conspiring that new metastasis would have appeared early after surgery, and the loss of strength due to surgery would have affected the ability to tolerate subsequent chemotherapy. Although minimally invasive surgery has increased the procedure's safety [12, 13], the loss of respiratory function after surgery significantly impacts the quality of life and decreases chemotherapy rates. In recent years, local consolidative therapy for oligometastasis and oligo-recurrence has attracted considerable attention in the field of cancer of various organs [14, 15]. The two-step evaluation may be useful in determining the indications for such treatment.

Our group had established an observation period of at least 3 months. Notably, the current study observed a linear increase in the incidence of new metastasis during the first year after PM diagnosis. If the observation period had been set to 1 year, 41% of the initially resectable cases would have developed new metastasis, with the absence of new metastasis during this period, indicating a low possibility of recurrence. Long-term observation raises some concerns, including lung tumour growth and PM spread to lymph nodes and downstream organs (brain, bone, etc.), as well as psychological factors among patients. However, in this study, a waiting period resulted in a slight increase in the maximum tumour diameter (1.2 ± 0.8–1.6 ± 0.9 cm), but the surgical procedure remained the same. Moreover, during the waiting period, none of the patients developed metastasis to downstream organs, such as the intrathoracic lymph nodes, bone or brain. Psychological factors are clinically important such that even after explaining the significance of the two-step determination, several patients remained anxious about waiting for a long period. A waiting period of over 3 months was determined after balancing the aforementioned factors and must be established appropriately depending on the target disease. Under our strategy, cases with a single peripheral metastasis that can be removed by wedge resection were operated on immediately after diagnosis because pathological diagnosis is vital for cases of solitary nodules, whereas wedge resection has little impact on reoperation even with recurrence. The results of this study showed a favourable prognosis for the immediate surgery group.

The question of whether chemotherapy should be provided during the follow-up period is a difficult one. Herein, 39% of the patients received chemotherapy after diagnosis, with no significant difference in the rate of early increase regardless of whether chemotherapy was provided. Meanwhile, multivariable analysis revealed that patients who received chemotherapy during observation had a poor prognosis. Although the decision on whether or not to administer chemotherapy was finalized during a multidisciplinary meeting, chemotherapy was probably recommended more aggressively for patients with a high recurrence risk, indicating the existence of a case selection bias. Although chemotherapy administered after PM diagnosis might have had certain inhibitory effects on early increase, its prognostic effect could not be determined in this study. The ongoing phase II trial, which studies the interactions of chemotherapy and metastasectomy in treating patients with PM from CRC, will provide valuable information [7].

### Limitations

This study has some notable limitations. This was a retrospective, observational and single-institution study. Analyses were exploratory in nature, and *P*-values may be interpreted as descriptive rather than confirmatory. This study included only patients referred to our department for PM from CRC. Therefore, our results can only be applied to generally healthy patients with controllable extrathoracic lesions. Although the decision on the indication for surgery was primarily based on whether new metastases were detected or not, it may also be influenced by various information, such as the treatment history for extrathoracic metastases, response to previous chemotherapy and general condition of the patients, which could lead to selection bias. Moreover, the time between PM diagnosis and the two-step decision on surgical indication was the immortal time. However, in reality, no patient died within 6 months from diagnosis in the no-surgery group. Therefore, the influence of immortal time bias in this study is considered minor. It is necessary to compare the prognosis of the surgical group with that of the nonsurgical group by randomized controlled trials to confirm whether patients with early increase should undergo surgery. However, because randomized controlled trials of surgery and non-surgery for PM are extremely difficult to conduct, we believe it is crucial to evaluate the results of clinical practice in observational studies. In our group, surgery is the first choice of local treatment for PM from CRC, and stereotactic ablative radiotherapy and radiofrequency ablation have been used in very few cases. A more aggressive selection of these ablative therapies may alter the treatment outcome and the range of indications for local treatment.

## CONCLUSION

Patients with colorectal lung metastases who developed new metastasis during the waiting period exhibited poor prognoses. Therefore, the two-step determination for indications of lung metastasectomy can be useful for identifying patients for whom curative surgery is impossible, especially among patients with multiple metastases.

## Data Availability

The data that support the findings of this study are available from the corresponding author upon reasonable request.

## Notes

Presented at the 29th European Conference on General Thoracic Surgery, 20–22 June 2021 (VIRTUAL).

## ABBREVIATIONS

CRC: Colorectal cancer
CT: Computed tomography
OS: Overall survival
PET: Positron emission tomography
PM: Pulmonary metastases

## References

[1] Parnaby CN , Bailey W, Balasingam A, Beckert L, Eglinton T, Fife J et al Pulmonary staging in colorectal cancer: a review. Colorectal Dis 2012;14:660–70. 21689294 10.1111/j.1463-1318.2011.02601.x

[2] Nanji S , Karim S, Tang E, Brennan K, McGuire A, Pramesh CS et al Pulmonary metastasectomy for colorectal cancer: predictors of survival in routine surgical practice. Ann Thorac Surg 2018;105:1605–12. 29518384 10.1016/j.athoracsur.2018.02.007

[3] Okumura S , Kondo H, Tsuboi M, Nakayama H, Asamura H, Tsuchiya R et al Pulmonary resection for metastatic colorectal cancer: experiences with 159 patients. J Thorac Cardiovasc Surg 1996;112:867–74. 8873711 10.1016/S0022-5223(96)70085-5

[4] Okumura T , Boku N, Hishida T, Ohde Y, Sakao Y, Yoshiya K et al Surgical outcome and prognostic stratification for pulmonary metastasis from colorectal cancer. Ann Thorac Surg 2017;104:979–87. 28577846 10.1016/j.athoracsur.2017.03.021

[5] Hashiguchi Y , Muro K, Saito Y, Ito Y, Ajioka Y, Hamaguchi T et al; Japanese Society for Cancer of the Colon and Rectum. Japanese Society for Cancer of the Colon and Rectum (JSCCR) guidelines 2019 for the treatment of colorectal cancer. Int J Clin Oncol 2020;25:1–42. 31203527 10.1007/s10147-019-01485-z PMC6946738

[6] Treasure T , Dunning J, Williams NR, Macbeth F. Lung metastasectomy for colorectal cancer: the impression of benefit from uncontrolled studies was not supported in a randomized controlled trial. J Thorac Cardiovasc Surg 2022;163:486–90. 33840470 10.1016/j.jtcvs.2021.01.142

[7] Antonoff MB , Sofocleous CT, Callstrom MR, Nguyen QN. The roles of surgery, stereotactic radiation, and ablation for treatment of pulmonary metastases. J Thorac Cardiovasc Surg 2022;163:495–502. 33838914 10.1016/j.jtcvs.2021.01.143

[8] Iida T , Nomori H, Shiba M, Nakajima J, Okumura S, Horio H et al Prognostic factors after pulmonary metastasectomy for colorectal cancer and rationale for determining surgical indications: a retrospective analysis. Ann Surg 2013;257:1059–64. 23001087 10.1097/SLA.0b013e31826eda3b

[9] Murakawa T. Past, present, and future perspectives of pulmonary metastasectomy for patients with advanced colorectal cancer. Surg Today 2021;51:204–11. 32857252 10.1007/s00595-020-02119-y

[10] Tanaka Y , Maniwa Y, Nishio W, Yoshimura M, Okita Y. The optimal timing to resect pulmonary metastasis. Eur J Cardiothorac Surg 2008;33:1135–8. 18396056 10.1016/j.ejcts.2008.03.002

[11] Yamada K , Ozawa D, Onozato R, Suzuki M, Fujita A, Ojima H. Optimal timing for the resection of pulmonary metastases in patients with colorectal cancer. Med (Baltim) 2020;99:e19144. 10.1097/MD.0000000000019144 PMC7478587 32118717

[12] Ichinose J , Kohno T, Fujimori S, Mun M. Locoregional control of thoracoscopic lobectomy with selective lymphadenectomy for lung cancer. Ann Thorac Surg 2010;90:235–9. 20609783 10.1016/j.athoracsur.2010.03.049

[13] Mun M , Nakao M, Matsuura Y, Ichinose J, Nakagawa K, Okumura S. Video-assisted thoracoscopic surgery lobectomy for non-small cell lung cancer. Gen Thorac Cardiovasc Surg 2018;66:626–31. 30062622 10.1007/s11748-018-0979-x

[14] Gomez DR , Tang C, Zhang J, Blumenschein GR Jr, Hernandez M, Lee JJ et al Local consolidative therapy vs. maintenance therapy or observation for patients with oligometastatic non-small-cell lung cancer: long-term results of a multi-institutional, Phase II, randomized study. J Clin Oncol 2019;37:1558–65. 31067138 10.1200/JCO.19.00201 PMC6599408

[15] Sonoda D , Matsuura Y, Kondo Y, Ichinose J, Nakao M, Ninomiya H et al Comparison of local therapy in patients with lung oligo-recurrence of non-small-cell lung cancer. J Surg Oncol 2021;123:1828–35. 33684232 10.1002/jso.26453

